# Nanocrystalline Apatites: Post-Immersion Acidification and How to Avoid It—Application to Antibacterial Bone Substitutes

**DOI:** 10.3390/bioengineering10020220

**Published:** 2023-02-07

**Authors:** Christophe Drouet, Nicolas Vandecandelaère, Anke Burger-Kentischer, Iris Trick, Christina G. Kohl, Tanja Maucher, Michaela Mueller, Franz E. Weber

**Affiliations:** 1 CIRIMAT, Université de Toulouse, CNRS, Toulouse INP, 31000 Toulouse, France; 2 Fraunhofer IGB, 70569 Stuttgart, Germany; 3 Center of Dental Medicine, Oral Biotechnology & Bioengineering, University of Zurich, 8006 Zurich, Switzerland

**Keywords:** nanocrystalline apatite, surface features, acidification effect, surface equilibration, antibacterial bone substitute, copper, silver, calvaria

## Abstract

Biomimetic nanocrystalline apatites analogous to bone mineral can be prepared using soft chemistry. Due to their high similarity to bone apatite, as opposed to stoichiometric hydroxyapatite for example, they now represent an appealing class of compounds to produce bioactive ceramics for which drug delivery and ion exchange abilities have been described extensively. However, immersion in aqueous media of dried non-carbonated biomimetic apatite crystals may generate an acidification event, which is often disregarded and not been clarified to-date. Yet, this acidification process could limit their further development if it is not understood and overcome if necessary. This may, for example, alter biological test outcomes, during their evaluation as bone repair materials, due to potentially deleterious effects of the acidic environment on cells, especially in in vitro static conditions. In this study, we explore the origins of this acidification phenomenon based on complementary experimental data and we point out the central role of the hydrated ionic layer present on apatite nanocrystals. We then propose a practical strategy to circumvent this acidification effect using an adequate post-precipitation equilibration step that was optimized. Using this enutralization protocol, we then showed the possibility of performing (micro)biological assessments on such compounds and provide an illustration with the examples of post-equilibrated Cu^2+^- and Ag^+^-doped nanocrystalline apatites. We demonstrate their non-cytotoxicity to osteoblast cells and their antibacterial features as tested versus five major pathogens involved in bone infections, therefore pointing to their relevance in the field of antibacterial bone substitutes. The preliminary in vivo implantation of a relevant sample in a rat’s calvarial defect confirmed its biocompatibility and the absence of adverse reaction. Understanding and eliminating this technical barrier should help promoting biomimetic apatites as a genuine new class of biomaterial-producing compounds for bone regeneration applications, e.g., with antibacterial features, far from being solely considered as “laboratory curiosities”.

## 1. Introduction

Bone is a composite tissue essentially composed of collagen fibers mineralized by plate-like calcium phosphate nanocrystals exhibiting the apatite structure [1,2,3]. A wealth of information is now available in the literature to describe nanocrystalline apatite physicochemistry, whether of biological or synthetic origin. It is now admitted that apatite nanocrystals are constituted by an apatitic core (itself nonstoichiometric) covered by a disordered ionic hydrated layer in which the ions are relatively labile and located in non-apatitic chemical environments [1,4,5,6]. Such non-apatitic features are clearly discernible using spectroscopy techniques such as FTIR or solid-state NMR, e.g., in the case of HPO_4_^2−^ ions that represent the main speciation of phosphate ions (as opposed to PO_4_^3−^) within the nanocrystal surface layer [1,6]. This hydrated ionic layer is an interphase domain fully belonging to the nanocrystal edifice [4,7], but it is in contact with the precipitating medium. Its non-apatitic character can be seen as a reminiscence of the status of the ions from the original precipitating medium (either body fluids in vivo or the precipitation medium in vitro), which is linked with the limited kinetics of hydroxyapatite crystallization at a moderate temperature such as at 37 °C. Therefore, any interaction of the nanocrystals with their surrounding medium will in fact imply a direct interaction with this non-apatitic surface layer. Hereby, any protein, cell, or adsorbed drug interacting with a biomimetic apatite nanocrystal will necessarily interact *first* with this amorphous-like surface layer and not with the apatitic core of the crystals. It may be reminded that the depth of this layer may be far from negligible at the molecular scale, being estimated from NMR studies to a few nanometers (for a moderate maturation state of the apatite phase) [4,8]. Since this layer is a crucial feature of bone-like apatite nanocrystals, the development of biomaterials involving such compounds obviously necessitates good knowledge of its possible interactions with its surroundings.

The immersion of calcium-deficient/nanocrystalline apatites was sometimes reported to lead to unexplained acidification of the medium, which may negatively impact their behavior after implantation [9]. This effect is likely linked to the surface features of apatite nanocrystals, but this question has not yet been specifically addressed. Additionally, in the realization of in vitro (micro)biological tests, which are carried out in static conditions, this acidification effect could lead to altered results due to the undesirable effects of acidic pH on mammalian cells or bacteria.

In this contribution, we first experimentally illustrate this acidification event by immersing dried nanocrystalline (non-carbonated) apatite particles in representative culture media. We then refer to the “hydrated layer model” to elucidate the origins of this acidification process and propose a related mechanism at the molecular scale. Finally, we investigate practical equilibration methodologies to avoid this phenomenon and propose an optimized approach to do so. We ultimately validate this approach with selected in vitro evaluations on relevant cells and bacterial strains to illustrate the possibility of developing bioactive materials, with the example of copper- and silver-substituted nanocrystalline apatites to convey antibacterial properties for bone repair applications.

## 2. Materials and Methods

### 2.1. Samples Synthesis and Physicochemical Characterization

Bio-inspired nanocrystalline apatite (non-carbonated) was precipitated at room temperature (RT ≅ 20 °C) by mixing a calcium nitrate Ca(NO_3_)_2_ · 4H_2_O solution (0.3 M, Merck Emsure, Darmstadt, Germany) and a di-ammonium hydrogenphosphate (NH_4_)_2_HPO_4_ solution (0.6 M, Merck Emsure, Darmstadt, Germany). The excess of phosphate ions relative to the composition of hydroxyapatite was chosen here as a way to maintain an internal pH buffering effect, close to the physiological value (pH ≅ 7.4) without the need of adding in the medium external species such as tris(hydroxymethyl)aminomethane (TRIS). After mixing and stirring for 5 min, the precipitating medium was left to mature for the desired time (e.g., 1 day, as indicated in the text, leading to the sample denoted hap-1d) before filtration on the Büchner funnel and thorough washing with deionized water (unless otherwise mentioned). The washed precipitate was then typically freeze-dried (−80 °C, 10 mbar).

The copper- (Cu^2+^) and silver- (Ag^+^) doped biomimetic apatites were also prepared to convey antibacterial properties. The synthesis protocol was similar to the one mentioned above for non-doped biomimetic apatite, with a maturation time of 1 day, but replacing a certain molar percentage of Ca^2+^ ions in the precipitating medium—as indicated in the text—by the equivalent amount of copper nitrate (Cu(NO_3_)_2_ · 3H_2_O, Acros Organics, Geel, Belgium) or silver nitrate (AgNO_3_, Alfa Aesar, Kandel, Germany), respectively. In the case of copper doping, the (NH_4_)_2_HPO_4_ reactant was replaced by its sodium counterpart Na_2_HPO_4_ (Merck Emsure, Merck Emsure, Darmstadt, Germany) based on a previous study showing easier reproducibility (unpublished data). The doping rates in Cu^2+^ and Ag^+^ will be provided in the text in terms of mol.% of doping ion compared to Ca^2+^ in the starting precipitation medium.

When mentioned in the text, the stoichiometric hydroxyapatite (HA) Ca_10_(PO_4_)_6_(OH)_2_ and apatitic tricalcium phosphate (TCP_ap_) Ca_9_(PO_4_)_5_(HPO_4_)(OH) (in which all the HPO_4_^2−^ ions are localized in apatitic chemical environments) were used for comparative purposes. The HA was obtained by adding a solution of ammonium phosphate into a boiling solution of calcium nitrate, in stoichiometric proportions and in the presence of ammonia. The precipitate was filtered, washed, dried, and then calcined at 1000 °C for 1 h. The TCP_ap_ was prepared in hydrothermal conditions (use of a sealed hydrothermal reactor) by hydrolyzing β-TCP to 260 °C for 16 h, in water.

From the obtained freeze-dried powders, pellets (diameter: 13 mm, depth: 1.1 mm) were prepared, when necessary, using uniaxial pressing (Hounsfield H25K-S press, Hounsfield at RT, typically under 80 MPa and using 150 mg apatite powder per pellet).

The XRD, FTIR, and chemical analyses confirmed the nanocrystalline and apatitic character of the samples in a previous study^1^. Here, additional FTIR analyses (Nicolet 5700, Thermo Fisher Scientific, Waltham, MA, USA) in the mid-IR spectral range: 400–4000 cm^−1^ and with a resolution of 4 cm^−1^ were performed to complement the vibrational characterization of the samples. The spectral decomposition in the ν_3_PO_4_ domain was performed as previously detailed [1] to follow in a semi-quantitative way the evolution of HPO_4_^2−^ and OH^−^ contents within the apatite compounds. ICP-OES analyses were performed (Ultima 2, Horiba, Kyoto, Japan) for calcium and phosphorus titration of ions released upon the immersion of dried apatite samples (λ_Ca_= 317.933 nm, λ_P_ = 214.914 nm). These experiments were performed at the *Cellule Mesure et Analyse* center, LGC laboratory, Toulouse. The FEG-SEM observations were performed using a LEO 1530 VP microscope (Carl Zeiss, Iena, Germany) operated at 5 kV.

The ion release tests from the doped apatite powders (150 mg) were carried out in static conditions in pure deionized water (50 mL) at RT over 6 days. Then, the medium was filtered to determine the amount of released doping ions. The Ag^+^ ions were titrated using spectrophotometry at λ = 560 nm using a DR2010 (HACH, Loveland, CO, USA) kit and following the provider’s specifications.

### 2.2. Acidification Follow-Up

The acidification phenomenon was experimentally evaluated by contacting the hap-1d pellets with culture media in 24-well culture plates. The qualitative pH variations were created by contacting each pellet with a 1 mL DMEM medium containing phenol red as the pH indicator (DMEM, 4.5 g/L D-glucose, + L-glutamine, Gibco by Life Technologies (Thermo Fisher Scientific, Waltham, MA, USA), supplemented with 2 mM glutamine (Stock 200 mM), 100 U/mL penicillin + 100 μg/mL streptomycin (Stock: 10,000 U/mL Pen, 10,000 μg/mL Strep), 1% insulin, transferrin, and selenium (Stock: 100x ITS-G) and 10% fetal calf serum) at 37 °C and for 24 h. The DMEM medium contains a sodium bicarbonate (NaHCO_3_) buffer system (3.7 g/L) and therefore requires a from 5 to 10% CO_2_ environment to maintain the physiological pH. However, the acidification follow-up was noticed whether the incubation was created in a cell culture incubator with 5% CO_2_ or in a regular atmosphere. The quantitative pH recording was carried out in 2 mL αMEM medium (Gibco by Life Technologies, Thermo Fisher Scientific, Waltham, MA, USA) containing a sodium bicarbonate (NaHCO_3_) buffer system (2.2 g/L) as well as phenol red as a pH indicator at 37 °C and for 7 h under atmospheric air. An additional test was carried out to investigate the role of the mass of the immersed hap-1d sample, by subjecting it to 20 mL of deionized water at pH 7.4.

### 2.3. Post-Equilibration (Enutralization) Procedure

In order to neutralize the observed acidification phenomenon, different washing solutions were tested during the filtration step directly on the Büchner funnel: (a) water/NaOH solutions at pH 9 and 11, (b) water/Na_2_HPO_4_ solution at pH 9, and (c) water/Na_3_PO_4_:12H_2_O solution at pH 10 and 11. A total of 500 mL of these washing solutions was used by adding aliquots of 100–150 mL and pausing for a few minutes between each addition. In all cases, a final washing step using pure deionized water (200 mL) was then performed prior to freeze-drying.

### 2.4. Cytotoxicity Evaluations

For the cytotoxic assays, we used the adherent human osteosarcoma cell line CAL-72 (DSMZ no.: ACC 439), which is generally known to be closely related to normal osteoblasts. The neutral red (NR) colorimetric assay was chosen to measure the viability of the cells in response to the apatite samples. This cell viability test is based on the ability of viable cells to incorporate and bind neutral red within lysosomes. NR is a cationic dye that readily penetrates the cell membrane and intracellularly accumulates in lysosomes, where it binds to anionic sites of the lysosomal matrix. The changes of the cell surface or the sensitive lysosomal membrane lead to lysosomal fragility and other changes that gradually become irreversible. These alterations result in a decreased uptake and binding of NR. It is thus possible to distinguish between viable, damaged, or dead cells. The quantity of dye incorporated into cells is measured using spectrometry and is directly proportional to the number of cells with an intact membrane of viable cells to incorporate and bind Neutral Red (NR) within lysosomes. Based on the ability of viable cells to incorporate and bind Neutral Red (NR) within lysosomes, the changes of the cell surface or the sensitive lysosomal membrane result in a decreased uptake and binding of NR.

#### 2.4.1. Assay Procedure

For cytotoxic analysis, the powder of the apatite samples was formed into round pellets (12 mm diameter and 1 mm thickness) using unixial pressing (130 mg/pellet) and the pellets were sterilized using gamma irradiation (BBF GmbH, Kernen-Rommelshausen, Germany). For the apatite pellets, 160 μL of an isotonic NaCl solution (0.9% NaCl) were added for 30 min. After that, the system was incubated for 24 h at 37 °C and 5% CO_2_ with 500 μL cell culture media (DMEM, 4.5 g/L D-glucose, + L-glutamine, Gibco by Life Technologies). This was supplemented with 2 mM glutamine (Stock 200 mM), 100 U/mL penicillin + 100 μg/mL streptomycin (Stock: 10,000 U/mL Pen, 10,000 μg/mL Strep), 1% insulin, transferrin and selenium (Stock: 100x ITS-G), and 10% fetal calf serum. All the supplements were also provided by Gibco by Life Technologies.

#### 2.4.2. Preparation of the Test Cell Line

For adhesion, 30 000 cells of the CAL-72 cell line were seeded in a 96-well micro-titer plate (MTP) and incubated in a cell incubator at 37 °C and 5% CO_2_ in a humidified atmosphere overnight. In the next step, the prepared supernatant samples (see Section 2.4.1) were applied undiluted and diluted at 1:3.33. After an incubation time of 24 or 48 h as specified, we carried out the Neutral Red development and finally the detection using photometry and microscopy. The quantity of the NR dye incorporated into the cells was measured using spectrometry at 540 nm and was directly proportional to the number of cells with an intact membrane. The experiments were performed in quadruplicate (n = 4) and the results are provided in terms of mean ± standard deviation.

### 2.5. Antibacterial Activity

The antibacterial activities of non-doped, Cu-doped, and Ag-doped biomimetic apatite samples ere evaluated versus 5 selected bacterial strains relevant to bone infections, namely *Staphylococcus aureus* DSM 20231, *Staphylococcus epidermidis* DSM 20044, *Escherichia coli* MG1655 DSM 18039, *Pseudomonas aeruginosa* PAO1 DSM 22644, and *Actinomyces denticolens* DSM 20671.

#### 2.5.1. Pre-Cultivation of Bacterial Strains

The aerobic bacterial strains (*S. aureus*, *S. epidermidis*, *E. coli*, and *P. aeruginosa*) obtained from the DSMZ were starved as equivalents of 0.5 mL by freezing at −80 °C and re-cultivated on the LB-medium for further investigations at 37 °C in Erlenmeyer flasks by continuously shaking in a laboratory shaker for an oxygen supply for 12 to 24 h depending on the microorganism. The pH value was 7.0 ± 0.2. The anaerobic strain *A. denticolens* obtained from the DSMZ was starved as equivalent of 0.5 mL by freezing at −80 °C and re-cultivated on a modified PYG-Medium at pH 7.2 (see DSMZ medium No. 104, where the soluble oxygen was displaced by gassing with a mixture of 80% H_2_ and 20% CO_2_ for 30 min) for further investigations at 37 °C using an anaerobic jar with an atmosphere of 80% H_2_ and 20% CO_2_ for 24 h.

The bacterial growth was monitored by measuring the optical density (OD) at 600 nm as well as counting the colony forming units (cfu) on appropriate agar plates. The microorganisms were harvested using centrifugation and suspended in a fresh growth medium in micro-titer plates with sterilized apatite samples for biofilm formation, with an incubation temperature of 37 °C.

#### 2.5.2. Antibacterial Testing

As the evaluation of the biofilm formation and the bacterial growth was essential to the study, the round pressed samples of 12 mm diameter and 1 mm thickness were used. The samples were transported in 24-well plates and sterilized using gamma irradiation (BBF GmbH, Kernen-Rommelshausen, Germany).

#### 2.5.3. Standardized Method for Comparison of Anti-Bacterial Properties

Every experiment was conducted with the first step of cultivating the relevant bacterial strains overnight. The reproducible results were ensured using the same optical density (OD) of 1. Of note, even with the same OD, the absolute cell number somewhat differs within the strains due to the cell size and OD is not strictly linearly related to the cell count except within a limited range. Thus, the more reliable way to obtain reproducible conditions is by using cfu counting. Yet, precedent experiments of the correlation of OD and cfu showed that, despite the differences in size and shape of the bacterial strains, the number of cfu are within the same range. This bacterial solution was applied on the apatite pellets placed in an MTP. An incubation period of 24 h at 37 °C followed. The experiments were performed in triplicate (n = 3) and the results are provided in terms of mean ± standard deviation.

### 2.6. In Vivo Implantation

#### 2.6.1. Animal Model

In view of implantation in rat calvaria, skeletally mature, female rats (age 3 months, weight 200 g) were sedated and further anaesthetized using a Halothan-N_2_O inhalation method. Four rats were used per tested group. The surgical area was clipped and prepared with iodine for aseptic surgery. A linear incision was cut from the nasal bone to the mid-sagital crest. The soft tissues were reflected and the periosteum was dissected from the site (occipital, frontal, and parietal bones) by scraping the calvarial bones with a scalpel. After placement of the materials, the soft tissues were sutured. Analgesia was provided by injection of Novalgin (50 mg/kg). The animals were sacrificed by CO_2_ at day 28; the calvarial bone, including the skin, was excised to not disturb the tissues. The standard ethical rules were scrupulously followed all along the study.

#### 2.6.2. Histology

The samples were first prepared with a sequential water substitution process that involved 48 h in 40% ethanol, 72 h in 70% ethanol (changed 24 h), 72 h in 96% ethanol, and finally 7 h in 100% ethanol. The samples were placed in xylene for 72 h for defatting the recovered bone (changed every 24 h). Next, infiltration was performed by placing the samples in methyl methacrylate (MMA) for 72 h (Fluka) followed by 3 days in 100 mL MMA + 2 g dibenzoyl peroxide (Fluka), at 4 °C. The samples were embedded by placing them in 100 mL MMA + 3 g dibenzoyl peroxide + 10 mL plastoid N or dibutyl phthalate (Merck) and allowing polymerization to occur at 37 °C in an air-tight water bath. The sections were prepared. Upon polymerization, the samples were ground to 25–50 µm thickness and the total proteins within the tissues were stained using Coomassie brilliant blue to ensure that all the tissue remained undisturbed. Digital images were taken using an image analysis program (Adobe Photoshop, version 2012 CS6 (13.0), San Jose, CA, USA).

## 3. Results and Discussion

**Figure 1:**
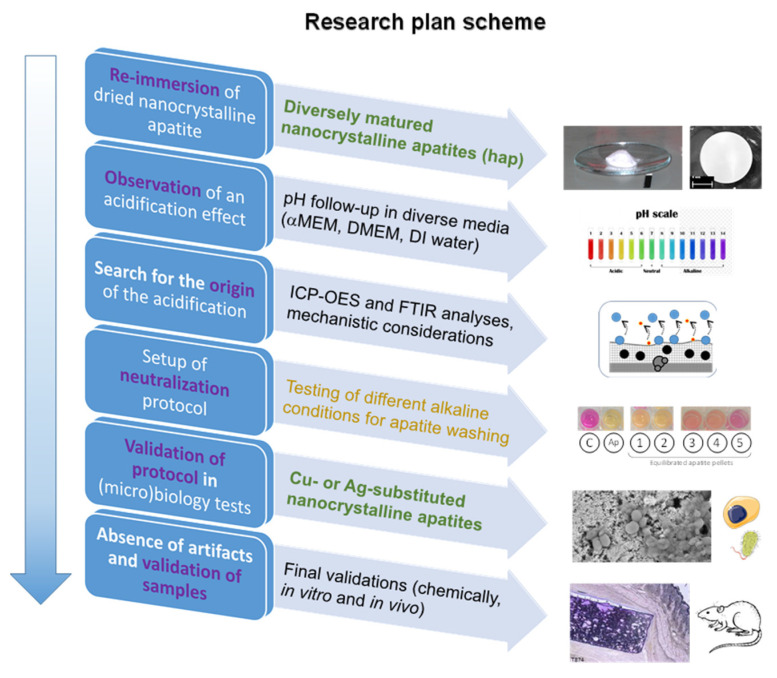
Overview of the research plan followed in this study.

This study originates from observations we gathered from preliminary in vitro trials on *S. aureus*, *S. epidermidis*, *E. coli*, and *P. aeruginosa* bacteria in the presence of non-carbonated nanocrystalline apatites (e.g., matured I day and hap-1d), showing the killing of all bacteria with a concomitant acidification of the culture medium qualitatively noticed from the color modification of the phenol red pH indicator. These findings unveiled such “false positive” results as the assessment of antibacterial properties could potentially only be due to the change of the medium pH, which is deleterious to the bacterial cells regardless of the substrate in contact. In order to shed light on these observations, reminding remarks reported by other authors [9], not yet elucidated, the present general study was performed with a multifold intention: (1) to quantitatively follow this pH evolution, (2) to investigate its chemical origin, (3) to set up a remediation protocol to neutralize this acidification effect, and (4) to validate the efficacy of this neutralization methodology. For the latter, we decided to illustrate this validation on “neutralized” Ag- and Cu-doped nanocrystalline apatites, with the view to show their promise as antibacterial bone substitutes by way of both in vitro and in vivo evaluations. Figure 1 provides a schematic view of this research study.

### 3.1. Occurrence and Origin of an Acidification Event after First Re-Immersion of Dried Biomimetic Apatites

**Figure 2:**
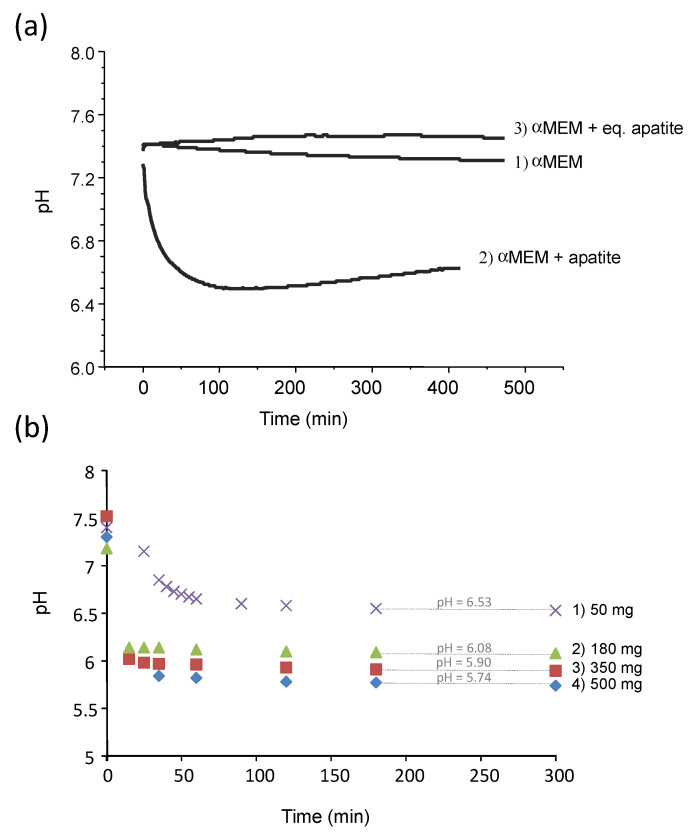
(**a**) pH evolution over time at 37 °C of αMEM medium, with or without re-immersed hap-1d apatite compound. The terms “eq. apatite” refer to sample hap-1d equilibrated at pH 11 in Na_3_PO_4_ · 12H_2_O during the washing step (see text); (**b**) pH evolution over time in deionized water (20 mL, initial pH 7.4) upon re-immersion of an increasing mass of hap-1d apatite compound.

The pH of the pure αMEM medium was monitored over 7 h at 37 °C, showing a stable pH value in the range from 7.4 to 7.3 (Figure 2a, curve 1), even in contact with atmospheric air (thus without the use of a cell culture incubator fed with CO_2_). In contrast, in the presence of the freeze-dried (non-carbonated) biomimetic apatite hap-1d (Figure 2a, curve 2) previously characterized in detail [1], the pH of the medium dropped significantly from 7.4 down to about 6.6 within the same time frame. A similar effect was noticed in other media such as DMEM. These results highlight the existence of interactions between apatite nanocrystals and the surrounding solution, leading to an acidification effect. As mentioned above, these observations indicate other authors’ reports that have yet to be explained so far [9]. In order to avoid any potential artefact due to a possible complexation process involving the ions and molecular species contained in αMEM, the immersion was reproduced in deionized water (pH set to 7.4 with NaOH/HCl). As in αMEM, a similar pH drop from 7.4 down to 6.53 was noticed again after immersing hap-1d (Figure S1 (in Annex—Supplementary Information), curve a). It may be noted that the pH stabilization occurred significantly faster in pure water than in αMEM, which may reasonably be related to the presence of molecular entities in αMEM delaying its kinetic evolution toward equilibrium.

To further investigate this acidification phenomenon, pH was also followed by immersing a nanocrystalline apatite sample matured for only 20 min (hap-20 min), thus exhibiting a significantly lower degree of maturation and a larger amount of the hydrated layer on the nanocrystal surface, as explained previously [1,10]. The stabilized pH for hap-20 min (Figure S1, curve b) then reached 6.41. This pH value refers to more acidic conditions than for hap-1d (all other conditions being the same). Additionally, the slope of the pH = f(t) curve was dramatically steepened for hap-20 min compared to hap-1d, as the stabilization was obtained after ~30 s for hap-20 min versus ~70 s for hap-1d. These findings indicate that the degree of acidification of the solution after re-immersion of the freeze-dried nanocrystalline apatites directly depends on their maturation state: the immersion of less matured samples (i.e., exhibiting a larger amount of the non-apatitic hydrated layer on the surface of the nanocrystals) generates a greater acidification effect.

The effect of the mass of dried apatite sample immersed in a constant volume (20 mL deionized water, initial pH 7.4) was also investigated for hap-1d (Figure 2b). As may be noticed, a clear tendency of increased acidification (stabilized pH values from 6.53 down to 5.74) was found upon increasing the amount of immersed freeze-dried sample. This phenomenon cannot be attributed to a regular dissolution behavior of a solid phase since no obviously acidic entity constituted the sample composition and it must be explained by a more complex surface/solution interaction mechanism. In addition, this phenomenon is very rapid, as is initiated from the very first seconds following re-immersion in an aqueous medium.

**Figure 3:**
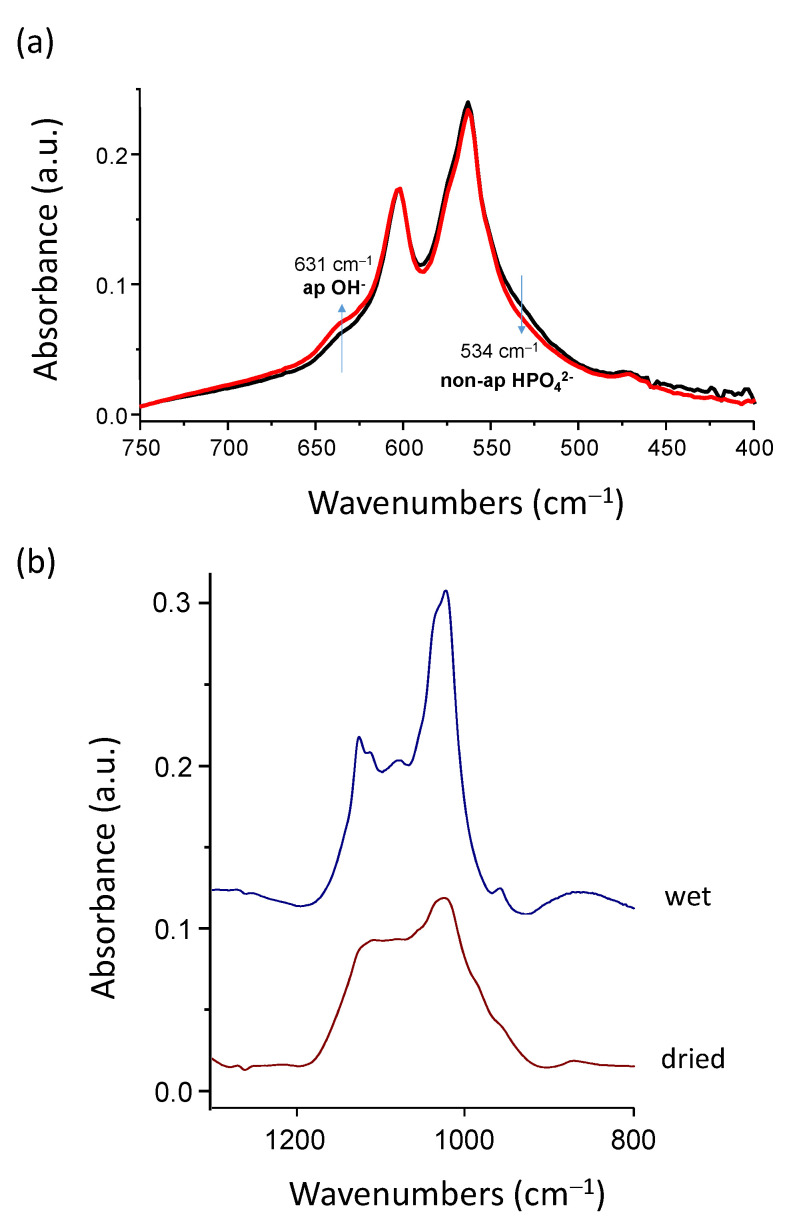
(**a**) FTIR spectra of the ν_lib_OH-ν_4_PO_4_ domain and evolution of non-apatitic HPO_4_^2−^ and apatitic OH^−^ contents (from spectral decomposition) before (black line) and after (red line) re-immersion of hap-1d in aqueous solution (initial pH 7.4, ~150 min re-immersion); (**b**) FTIR analysis of hap-20 sample in the wet and dry states in the ν_3_PO_4_ domain.

In order to explore the underlying mechanism of this acidification phenomenon, the FTIR analyses of freeze-dried hap-1d were carried out before and after sample immersion for ~150 min, which corresponds roughly to the stabilization delay of the pH drop (see Figure 2). This rather short period of immersion is also interesting as it avoids any significant post-maturation; thus, we may assign any observed feature to the acidification process itself. In particular, the ν_4_PO_4_ vibration domain was analyzed, using the spectral decomposition procedure detailed previously [1]. By comparison with the spectral features of the initial hap-1d freeze-dried powder, the FTIR results after re-immersion point out (Figure 3a) a decrease in the proportion of non-apatitic HPO_4_^2−^ ion content (band at ~534 cm^−1^). This evolution cannot be assigned to post-maturation due to the very limited duration of immersion (150 min) as compared to the initial maturation time (1 day). The immersion process thus directly promotes a noticeable decrease in surface HPO_4_^2−^ ions relative to other species. Since it is also accompanied by solution acidification, these results strongly suggest that some protonated phosphate ions are released into the solution at the time of re-immersion. Besides this decrease in non-apatitic HPO_4_^2−^ ion content, we can also note that the proportion of the band at ~632 cm^−1^ assignable to a libration band of apatitic OH^−^ ions also somewhat increases. This effect may again be explained by the release of some phosphate ions to the solution upon re-immersion, thus lowering the total amount of phosphate ions and, in turn, artificially increasing the relative proportion in the ν_4_PO_4_ domain of any other species active in IR, such as OH^−^.

The non-apatitic surface layer on biomimetic apatite nanocrystals contains a significant amount of protonated “non-apatitic” HPO_4_^2−^ phosphate ions [1]. In contrast, “non-apatitic” PO_4_^3−^ species are present only in a very limited amount at the surface of the nanocrystals [1,10]. In other words, the phosphate speciation at the surface is clearly favoring the protonated HPO_4_^2−^ form over PO_4_^3−^. The above FTIR findings have pointed to the release of protonated phosphate ions upon first re-immersion. However, the pK_a_ of the acid-base couple H_2_PO_4_^−^/HPO_4_^2−^ is 7.2, meaning it is, thus, very close to the physiological value. Therefore, at pH 7.4, the release of HPO_4_^2−^ ions from the solid phase would tend to consume (rather than release) some H^+^ from the solution to form a balanced amount of H_2_PO_4_^−^/HPO_4_^2−^ species, which is not observed here as evidenced by the pH drop. A plausible mechanism explaining both the acidification effect and the loss of protonated phosphate species as detected using FTIR would instead be based on the release of diprotonated H_2_PO_4_^−^ ions. Such ions could form upon interaction among adjacent HPO_4_^2−^ ions, via proton displacement (hopping). Such a hopping scenario can be schematized using the following scheme:

(1) $$2HPO_{4}^{}{{}^{2-}}_{solid\;phase}\to H_{2}PO_{4}{{}^{-}}_{solid\;phase}+PO_{4}{{}^{3-}}_{solid\;phase}$$

With the H_2_PO_4_^−^ ions formed at the solid top surface having two negative charges, they would become less attracted by surface Ca^2+^ cations than HPO_4_^2−^ (due to their less negative charge) and could then be more easily released upon re-immersion:

(2) $$H_{2}PO_{4}{{}^{-}}_{solid\;phase}\to H_{2}PO_{4}{{}^{-}}_{solution}$$

This release process is indeed in agreement with Christoffersen’s findings on apatite dissolution [11], stating that “when a negative surface ion has reacted with a H^+^ ion, all electrostatic bonds between that surface ion and the surrounding Ca^2+^ ions are weakened, and the activation energy needed to remove the negative surface ion is thus reduced”.

By releasing diprotonated H_2_PO_4_^−^ ions, an acidification effect would finally be expected due their tendency, when in solution in neutral conditions, to partly form HPO_4_^2−^ using the acid-base reaction:

(3) $$H_{2}PO_{4}^{}{{}^{-}}_{solution}{↔}_{}HPO_{4}{{}^{2-}}_{solution}+H^{+}{}_{solution}$$

For neutrality reasons, the release of negative H_2_PO_4_^−^ ions can only occur, however, if cations simultaneously leave the solid or if negative ions from the solution replace them. In pure water, the only conceivable option lies in the concomitant release of Ca^2+^ surface ions (one Ca^2+^ ion being released for two H_2_PO_4_^−^ ions to preserve electroneutrality).

In order to validate this mechanistic scenario, the calcium and phosphate ions released in solution upon re-immersion in water at initial pH 7.4 of hap-20 min, hap-3 h, and hap-1d samples (50 mg) were titrated using ICP-OES. The results (Table S1) confirmed our above hypothesis by evidencing the actual release of both calcium and phosphate ions and, as expected, the Ca/P ratios reached in solution were measured close to 0.50 (in the range 0.44–0.56). Therefore, these results strongly substantiate our hypothesis of the released dissolved species corresponding to the overall stoichiometry “Ca(H_2_PO_4_)_2_“ upon the re-immersion of dried nanocrystalline apatite powders.

All the above results point out that, upon the re-immersion of dried nanocrystalline apatites, the fast release of ions corresponding to the global stoichiometry Ca(H_2_PO_4_)_2_. It is not clear at this point whether Ca(H_2_PO_4_)_2_ “clusters” may have formed/accumulated on the crystal top surface during the drying step itself or if the involved ions are simply concomitantly released in the solution upon re-immersion. In any case, the drying step is likely to partially dehydrate the utmost surface layer, which may disorganize the ionic environment of top-surface ions. This partial loss of water is indeed expected to modify the H-bonding array within the hydrated surface layer. Additionally, the (large) oxygen atoms from the H_2_O surface molecules may, in the wet state, participate in the coordination sphere of non-apatitic surface ions and the water loss could then destabilize the top-surface arrangement of the ions. Note that the formation of a separate monocalcium phosphate phase, such as monohydrate (MCPM) or anhydrous (MCPA), has never been noticed using common methods (XRD, FTIR, Raman, and NMR) including by us. Only top-surface ions are probably implicated, thus limiting the detection of eventual Ca(H_2_PO_4_)_2_ clusters at the crystals surface even if they exist as such on the dried surface. In both hypotheses anyway (cluster or concomitant ion release), the phosphate ions should be released in their diprotonated form H_2_PO_4_^−^ to explain our observations (acidification, Ca/P ~0.5 in solution, and IR detection of a decrease in non-apatitic HPO_4_^2−^ content). This speciation is thought to arise from protons hopping from one HPO_4_^2−^ ion to the other, as described in Equation (1).

Our findings thus advocates that the acidification event observed after the first re-immersion of dried nanocrystalline apatites is linked to the alteration of the environment of top-surface ions from the surface layer. The re-immersion of other apatitic calcium phosphates where no hydrated layer is present on the crystal surface, such as stoichiometric hydroxyapatite (HA) Ca_10_(PO_4_)_6_(OH)_2_ or apatitic tricalcium phosphate (TCP_ap_) Ca_9_(PO_4_)_5_(HPO_4_)(OH) (in which all HPO_4_^2−^ ions are localized in apatitic chemical environments) was also followed for verification and, as anticipated, it did not lead to any significant pH change (Figure S2). This observation confirms that the acidification process is assignable to the non-apatitic surface layer present on biomimetic apatite nanocrystals.

**Figure 4:**
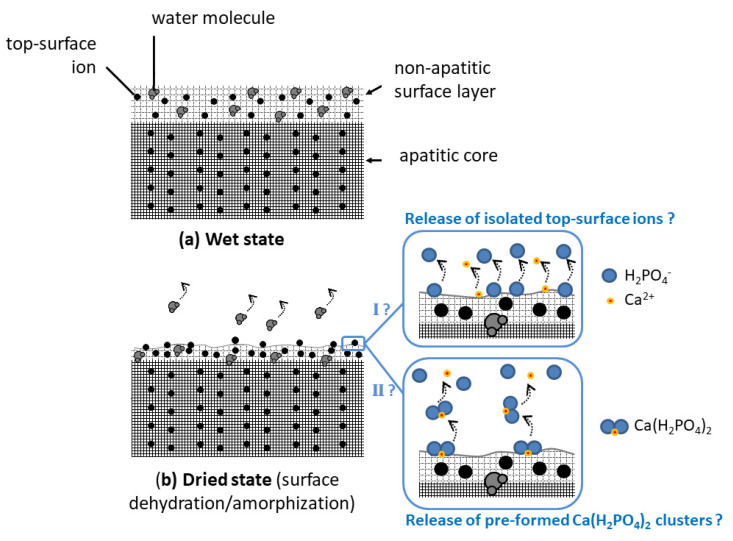
Schematic view of surface amorphization due to drying. The pathways I and II represent the two hypotheses discussed in the text to explain the acidification effect observed upon first re-immersion (I: release of isolated top-surface ions, II: release of pre-formed Ca(H_2_PO_4_)_2_ clusters).

In order to further inspect the effect of drying on the nanocrystals physicochemical features, FTIR analyses were performed on wet, freshly precipitated apatite nanocrystals. The analysis of wet samples with a short maturation time such as 20 min clearly showed the existence of a fine band structure especially visible in the ν_3_PO_4_ domain (Figure 3b). These fine features can be related to the specific organization of biomimetic apatite nanocrystals, exhibiting a non-apatitic hydrated ionic layer on their surface [12]. Upon drying, however, this fine IR structure becomes noticeably altered, leading to a smoothened envelope (Figure 3b). This loss of vibrational details can be explained by some additional surface “amorphization” due to the removal of some “structural” water molecules from the hydrated ionic layer. These observations favor the hypothesis of a partial denaturation of the ionic environment of utmost surface ions and they also agree with the easier release of surface ions upon re-immersion. This may be schematized as shown in Figure 4, where the two hypothetical pathways discussed above are illustrated: pathway I supposing the release upon the first re-immersion of isolated top-surface ions and pathway II supposing the release of the pre-formed Ca(H_2_PO_4_)_2_ clusters.

The above findings thus indicate that the (first) re-suspension of dried apatite nanocrystals in an aqueous medium is accompanied by an acidification phenomenon arising from the equilibration of the crystal’s surface with the solution, leading to an ultimate release of protons. The underlying mechanism is thought to originate from the protonated character of phosphate surface ions on apatite nanocrystals combined with top-surface alterations of ionic environments due to the additional amorphization effect generated by the drying process.

### 3.2. Circumventing this Acidification Effect

The above findings have pointed out the occurrence of an acidification effect upon re-immersion of dried nanocrystalline apatite samples: a mechanism was then established to explain all the experimental observations made (FTIR, ICP-OES, pH follow-up), based on the ultimate release of H_2_PO_4_^−^ ions along with their calcium counter-ions. This effect may thus be linked to the presence of high amounts of protons on the non-apatitic surface layer on the nanocrystals, and thus to a marked acidic character. Based on this understanding, one way to circumvent this acidification could then lie in an equilibration step under alkaline conditions prior to drying.

**Figure 5:**
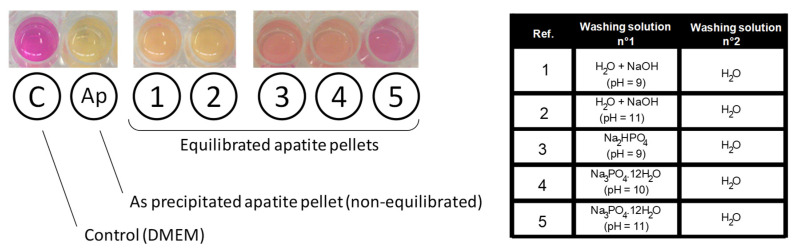
Coloration of phenol red indicator for various conditions. “C” denotes the control (DMEM alone), “Ap” denotes the freeze-dried apatite pellet (hap-1d, without equilibration), and refs. 1 to 5 denote experiments that included a modified washing step, with equilibration in different alkaline conditions. All experiments were performed at 37 °C for 24 h under atmospheric air, in 24-well culture plates (each well has an internal diameter of 15.6 mm).

In this view, the effect of different “equilibration” alkaline solutions during precipitate filtration was followed qualitatively by exposing each equilibrated apatite pellet to DMEM medium containing an internal pH indicator (phenol red), as summarized on Figure 5. A clear color transition of the pH indicator was observed from pink (for pure neutral DMEM at a pH 7.4) to light yellow for the reference apatite pellet exempt of equilibration washing step, thus evidencing a clear acidification effect. For equilibration washing carried out in aqueous solutions of NaOH (pH 9 and 11), an intermediate orange coloration was observed, indicative of an improvement (limitation of the acidification effect) but not totally satisfactory in these conditions. Even better results were obtained through equilibration in Na_2_HPO_4_ pH 9 and Na3PO4 · 12H2O pH 10 which led in contrast to red tones, thus indicating close-to-physiological pH value. Finally, the least color variation compared to the control was noticed for equilibration washing in Na3PO4 · 12H2O pH 11 (followed by final washing with pure deionized water). This last equilibration solution clearly led to the best result, with only a slight clearing of the pH indicator initial pink coloration but no signs of acidification, upon immersion of equilibrated apatite pellet. Experiments were repeated in αMEM to follow quantitatively the pH of the solution over 7 h (added in Figure 2a as curve 3). In this case, as may be expected from the results of Figure 5, the pH value remained stable around 7.4, thus confirming the absence of acidification effect in this case.

**Figure 6:**
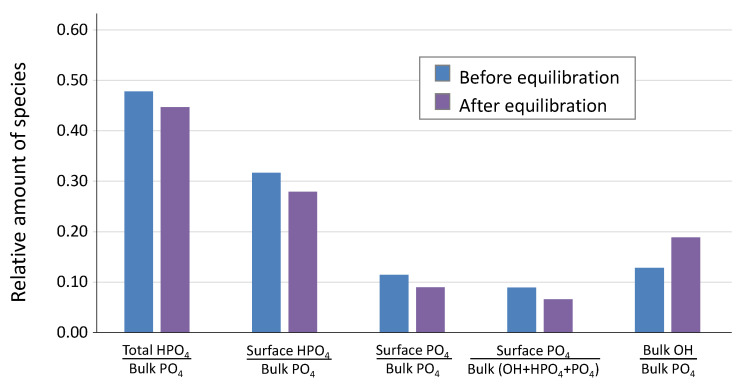
Evolution of the hydrated layer composition of sample hap-1d upon equilibration with Na3PO4 · 12H2O pH 11 (followed by final washing with pure deionized water). Data from FTIR spectral decomposition of the ν_lib_OH-ν_4_PO_4_ domain. The uncertainty on each value is evaluated to 0.03. Several ratios are shown involving apatitic (bulk) and non-apatitic (surface) ionic environments.

At this stage, it was interesting to investigate how the surface layer composition was modified by operating the equilibration step in the retained alkaline conditions. To this aim, FTIR decompositions in the ν_lib_OH-ν_4_PO_4_ domain were carried out before and after the equilibration/neutralization step (Figure 6), following the spectral decomposition methodology already described [1]. The proportion of the OH^−^ band appeared to increase in this spectral domain after the neutralization protocol, which is indicated by the increase in the [bulk OH/bulk PO_4_] ratio. Taking into account the high alkaline pH of 11, and the high reactivity of the freshly prepared apatite gel during this washing stage, this observation points to the increased filling of apatitic channels by additional OH^−^ ions during the alkaline treatment. The high concentration in OH^−^(aq) in the washing solution upon dissolution of the Na_3_PO_4_ · 12H_2_O salt can be deduced from the following equation, by taking into account that the nearest pK_a_ value is that of the couple HPO_4_^2−^/PO_4_^3−^ at 12.7, thus favoring the HPO_4_^2−^ speciation at pH 11:

(4) $$Na_{3}PO_{4}\cdot 12H_{2}O+H_{2}O\to 3Na^{+}+HPO_{4}^{2-}+OH^{-}+12H_{2}O$$

Additionally, the absence of acidification effect after re-immersion of dried apatite treated with this alkaline washing protocol strongly suggests that the density of surface protons (initially in the form of top-surface HPO_4_^2−^ ions) has coincidently decreased, which is corroborated by the decrease in the [surface HPO_4_/bulk PO_4_] ratio seen in Figure 6. A first simplistic view of this “surface neutralization” could initially be considered as follows:

(5) $$S-PO_{4}H^{2-}+OH^{-}\to S-PO_{4}{}^{3-}+H_{2}O$$

where S represents the top-surface of the crystals in contact with the solution. However, both the additional inclusion of OH^−^ in the crystals apatitic channels and Equation (5) would lead to an excess of negative charges in the solid and thus of positive charges in the solution. In order to maintain the overall neutrality of the solid and liquid phases, there is therefore a need to either incorporate simultaneously some cationic species into the solid or to expel from the solid some anionic species (necessarily of phosphate nature in this case). Residual Ca^2+^_(aq)_ are expected to be rare during this dynamic washing step, especially if an excess of phosphates was used in the precipitation stage. Although the incorporation of some sodium ions cannot be ruled out, it seems logical to expect that the release of top-surface phosphate entities could be primarily involved to comply with this electroneutrality rule. Indeed, it may be assessed from Figure 6 that both the [surface HPO_4_/bulk PO_4_] and the [surface PO_4_/bulk PO_4_] ratios decreased upon treatment. Then, Equation (5) should probably be modified into Equations (6) or (7) as follows—depending on the speciation of the released phosphate ion:

(6) $$S-PO_{4}H^{2-}\to S+HPO_{4}{}^{2-}$$

(7) $$S-PO_{4}H_{2}^{-}+OH^{-}\to S+H_{2}PO_{4}{}^{-}+OH^{-}\to S+HPO_{4}{}^{2-}+H_{2}O$$

where the $S-PO_{4}H_{2}^{-}$ species (involving H_2_PO_4_^−^ ions) would arise, as considered before, from proton hopping between two adjacent HPO_4_^2−^ ions due to the rather high mobility of surface protons in such nanocrystalline apatites.

In conclusion, the neutralization process appears to increase the hydroxylation of the nanocrystalline apatite while decreasing its surface acidity by removing surface protons, probably in the form of protonated phosphates to keep the electroneutrality of the system. This overall mechanistic scheme somewhat reminds the mechanism of apatite maturation proposed for nanocrystalline apatites in a previous paper [12], and could be seen here as an accelerated transient post-maturation in alkaline conditions. Due to its strong basicity, the alkaline solution of Na_3_PO_4_·12H_2_O pH 11 thus generates an environment propitious to neutralize the protonated acid phosphate species present within the hydrated layer to avoid acidification effects after drying and re-immersion. It may be remarked that the presence of phosphate ions in the medium upon dissolution of the Na_3_PO_4_·12H_2_O salt may additionally contribute to reinforce this overall effect by playing a buffering tendency towards the pK_a_ of the HPO_4_^2−^/PO_4_^3−^ acid-base couple, thus being more effective than simple washing with NaOH at pH 11 (Figure 5).

By preventing this pH drop—potentially detrimental to cells—the addition of this (simple) equilibration step in the preparation process of (non-carbonated) biomimetic apatites thus appears as an adequate method to circumvent this acidification artefact and allow producing readily usable biomimetic apatite-based biomaterials for bone applications.

### 3.3. In Vitro Cytocompatibility and Antibacterial Properties via Ion Doping

**Figure 7:**
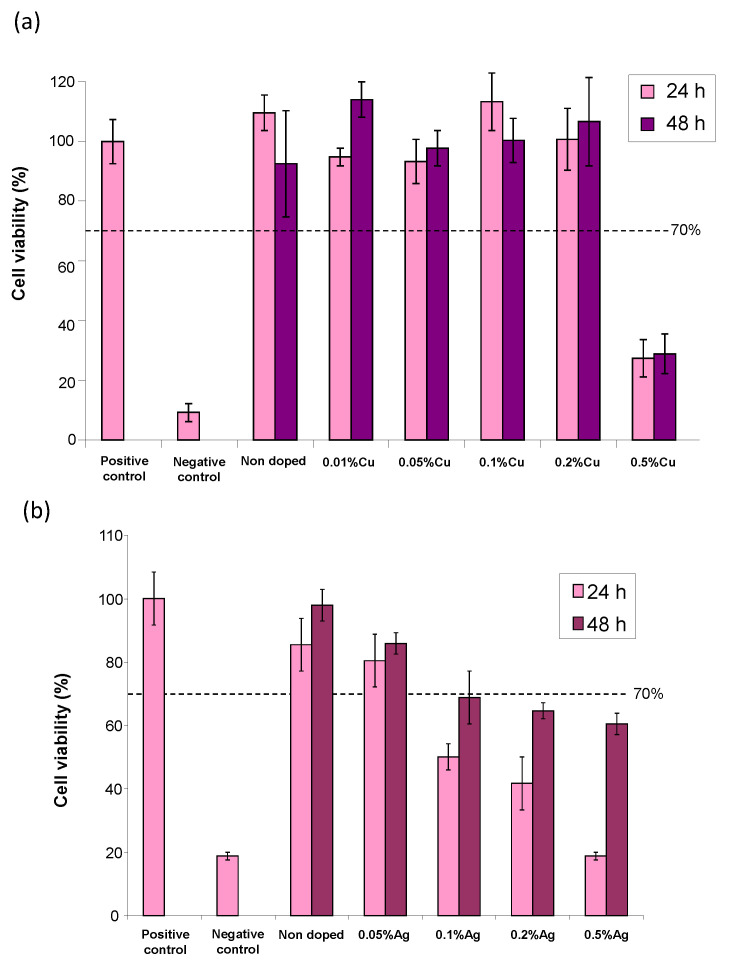
Cytotoxicity evaluation (CAL-72 human osteoblast cells) via the Neutral Red assay of pre-equilibrated hap-1d pelletized samples, non-doped (reference), or doped with either Cu^2+^ (**a**) or Ag^+^ (**b**) ions (percentages refer to the substitution of calcium in the precipitation medium) in our working conditions, relative to the controls. The cell viability % is provided with reference to the positive control set to 100%.

In order to validate these conclusions, in vitro tests have been carried out on equilibrated samples, with the view of showing their applicability while avoiding artifacts in the (micro)biological assessments. In a first stage, we studied human osteoblast (CAL-72) cell viability. The evaluation of the non-pre-equilibrated hap-1d sample unveiled some toxicity to the cells in our working conditions (*ca*. 40% cell viability, which is, thus, below the 70% limit considered as a toxicity threshold according to the ISO 10993-5:2009 standard), by evidencing a drop of the absorbance in the Neutral Red assay at t = 24 h (Figure S3). Taking into account the biomimetic character of such non-carbonated nanocrystalline apatites, this effect may reasonably be assigned to the acidification evidenced above upon the re-immersion of dried samples. A similar test was then carried out, after pre-equilibration during the washing step with the retained methodology using Na3PO4 · 12H2O pH 11 and water. In this case, as may be seen on Figure 7 (“non-doped” reference sample), the compound proved to be non-cytotoxic to CAL-72 osteoblasts cells, both at t = 24 and 48 h. These results clearly indicate the advantageous role of the pre-equilibration washing step, retaining the highly biocompatible compounds even in static conditions, in view of the setup of functional biomaterials.

One particularly appealing property to convey to such compounds intended as bone substitutes is antibacterial activity. This is especially important when accounting for the difficulty to eradicate established infections in bone tissue due to its highly porous character. Such antibacterial properties may be obtained by way of doping with ions such as Cu^2+^ or Ag^+^, which corresponds here to the substitute part of the Ca^2+^ ions by these doping ions at the time of synthesis. Antibacterial ions indeed have the advantage of avoiding bacterial resistance phenomena as opposed to antibiotics.

Copper ions are particularly appealing as they are already present in vivo (in metalloproteins) while they also exhibit other activities favoring bone regeneration such as pro-angiogenesis and osteoconduction. In this view, we have first prepared Cu^2+^-doped apatite samples with increasing doping rates and post-treated them using the optimized alkaline conditions mentioned above. The main physicochemical characteristics of these doped apatite samples were investigated and are reported on Figure S4. In particular, the “biomimetic” apatite nature of these samples was again assessed, with the physicochemical features close to those of the non-doped hap-1d and the actual doping rates found very close to the nominal rates in the precipitating solution.

The cytocompatibility with osteoblast cells of such post-treated Cu^2+^-doped apatites was then checked, first in a large range of Cu doping rates (Figure S5a) and then in a more narrow range of interest as reported in Figure 7a. As may be seen, except for the copper contents of 0.5 mol.% or higher, the samples with a lower Cu-doping rate led to cell viability well beyond 70% at both 24 and 48 h of contact, thus evidencing their non-cytotoxicity to CAL-72 cells.

**Figure 8:**
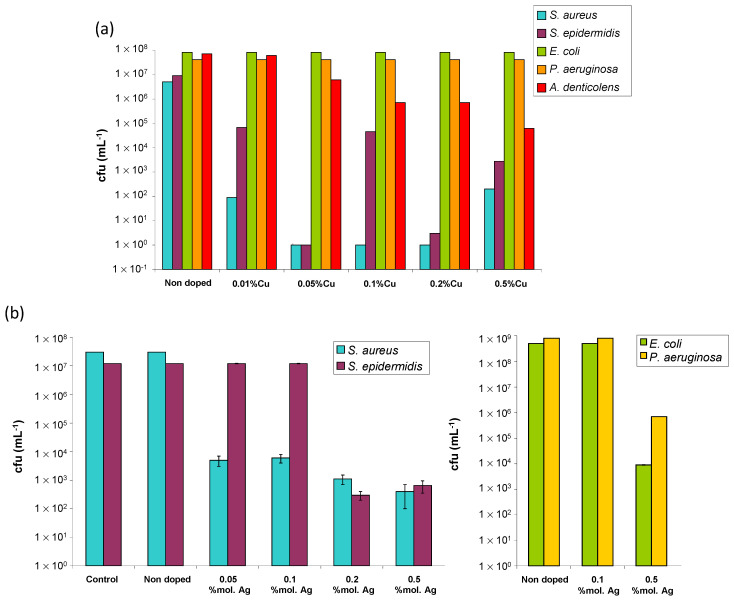
Antibacterial results of pre-equilibrated hap-1d pelletized samples, non-doped (reference) or doped with Cu^2+^ (**a**) or Ag^+^ (**b**) ions, versus different Gram-positive (*S. aureus*, *S. epidermidis*, and *A. denticolens*) or Gram-negative (*E. coli* and *P. aeruginosa*) bacteria, after 24 h of contact.

In a second step, the intrinsic antibacterial properties of these post-treated Cu-doped apatite samples were investigated on five selected bacterial strains relevant to bone infections. Four of them are aerobic bacteria, namely *S. aureus*, *S. epidermidis*, *E. coli*, and *P. aeruginosa*, and one is an aenaerobic bacterium, namely *A. denticolens* (Figure 8a). While the referenced non-doped sample did not show any intrinsic antibacterial activity, as expected, the samples doped with copper ions showed antibacterial properties with a dependence on the type of microorganism and on the dose of antibacterial agent used.

Such Cu-doped bio-inspired apatites were found to be particularly active on Gram-positive bacteria as *S. aureus*, *S. epidermidis*, and, to some extent, *A. denticolens*. For *S. aureus*, a significant decrease in bacterial colonies (exponential scale) was found for all samples, from a copper content as low as 0.01 mol.% (relative to calcium), thus leading to the elimination of more than 99.99% of the germs. For *S. epidermidis*, a similar trend was also found, but with a more noticeable dose-dependency. The samples with 0.01 and 0.1% of copper eliminate 99.00% of bacteria, whereas the samples with 0.5% copper eliminate 99.95% and those with copper contents between 0.05 and 0.2% eliminate more than 99.99% of the bacteria (the measure realized on the sample corresponding to 0.1% is probably artefactual). Therefore, the samples with copper contents between 0.05 and 0.2 mol.% relative to calcium are the most efficient for fighting against *S. epidermidis*. For *A. denticolens* (anaerobic), our results reveal a progressive decrease in the number of bacterial colonies as the copper content increases. If the sample with 0.01% copper appears rather inactive, the tested samples corresponding to higher copper contents (between 0.05 and 0.5% copper) lead to a decreased quantity of bacteria, with a reduction of 99.90% of the number of bacteria for a copper content of 0.5%. In contrast, for the Gram-negative *E. coli* and *P. aeruginosa* bacteria, the results obtained here did not show a significant antibacterial activity for our Cu-doped specimens as compared to the non-doped apatite.

**Figure 9:**
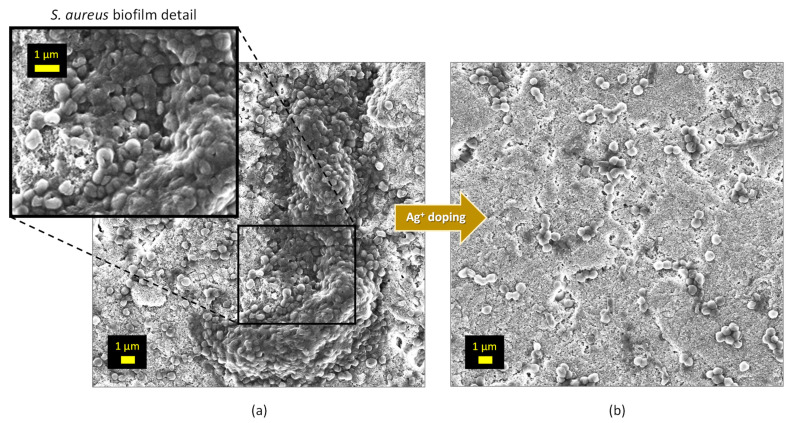
SEM observation of *S. aureus* (cocci) biofilm inhibition via Ag^+^ doping in biomimetic apatite (pellets): (**a**) non-doped apatite; (**b**) hap-1d 4% Ag apatite. The inlet in (**a**) shows a zoomed view of the biofilm formed on the non-doped sample.

Besides copper doping, the Ag^+^-doped samples were similarly prepared and tested. The main physicochemical characteristics of these silver-bearing apatite samples were investigated (Figure S4). As previously mentioned, the apatites close to hap-1d non-doped and exhibiting a biomimetic character were obtained. The possible incorporation of Ag^+^ in apatites has been reported on several occasions [13,14,15], including by some of us recently [16]. It may, however, be remarked that the experimental doping rates found for such Ag-doped compounds were lower than the nominal ones (i.e., used in the precipitation medium). This is probably linked to the different size and charge of the Ag^+^ ions compared to Ca^2+^, which require structural and compositional changes in the apatitic phase [15,16]. In vitro assays were again conducted on CAL-72 osteoblastic cells. A progressive decreasing tendency in cell viability was observed upon increasing the Ag doping, as may be seen in Figure S5 for a large Ag doping range. The refined results (Figure 7b) indicate that the samples with a nominal content of Ag^+^ of up to 0.5 mol.% (with reference to calcium) keep exhibiting a cell viability close to 80% at 48 h, unveiling their low toxicity. Antibacterial testing was also performed and the Ag^+^-doped samples proved to significantly lower than the amount of bacterial colonies (Figure 8b), for example, with a drop of 4 orders of magnitude for Gram-positive *S. aureus* and *S. epidermidis* for 0.1 and 0.2% Ag and a drop between 3 and 5 orders of magnitude for Gram-negative *E. coli* and *P. aeruginosa*. Thus, contrary to the Cu-doped samples studied in this work, the Ag-doped specimens were found to be both active against Gram-negative and Gram-positive bacterial strains. An SEM illustration of the anti-biofilm effect of Ag^+^ doping in biomimetic apatite is for example provided in the case of the *S. aureus* in Figure 9. As may be seen, an organized biofilm is clearly visible on the non-doped sample while silver doping allows for drastically limiting the number of bacteria present on the surface and the development of an organized biofilm.

**Figure 10:**
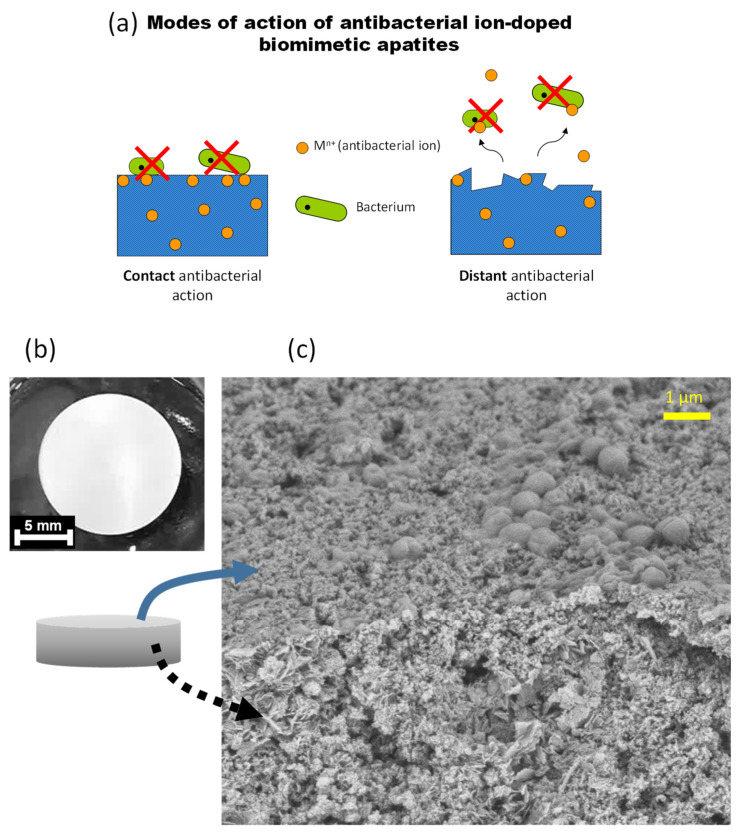
(**a**) Schematic of the antibacterial modes of action expected for ion-doped biomimetic apatites; (**b**) example of apatite pellet, and (**c**) observation of the localization of *S. aureus* bacteria on the top surface of a (non-doped) apatite pellet.

This antibacterial effect can presumably be conferred via both a “contact” mode of action (implying doping ions are exposed at the surface of the apatite crystals) and a “distant” phenomenon via the release of the antibacterial doping ions, as schematized in Figure 10a. To explore the eventuality of this second option, the release of Ag^+^ ions was followed from two Ag-doped apatite samples (hap 5% Ag matured for 20 min and 6 days) used as typical examples, in pure deionized water at RT. For both samples, about 1.2 mol.% of silver ions have been released after 6 days of immersion in simple static conditions even without cell activity, thus confirming that distant antibacterial phenomena may potentially occur. The general localization of microorganisms on typical pellets was also examined using FEG-SEM. As shown on the illustrative example provided in Figure 10b,c, the bacteria (*S. aureus* in this case) that were in contact with a hydroxyapatite pellet were found to be primarily present at the surface and not in the intercrystalline porosity. These findings further stress the relevance of a system capable of exhibiting both a “contact” and a “distant” antibacterial effect, as is the case for the ion-doped apatite samples prepared in this work.

As an intermediate summary on this part, noticeable antibacterial effects were evidenced in this work upon Cu^2+^ or Ag^+^ doping of biomimetic apatites equilibrated during the washing step with our optimized alkaline treatment. These effects could be detected (on Gram-positive strains for Cu^2+^ and on both Gram-positive and Gram-negative species for Ag^+^) at low doping rates and no “false” negative or positive data were noticed as no acidification was observed in particular for the non-doped reference sample. These findings open relevant perspectives in the field of bone repair and infection control while avoiding any acidification phenomena that are potentially detrimental to cells.

### 3.4. In Vivo Biocompatibility

**Figure 11:**
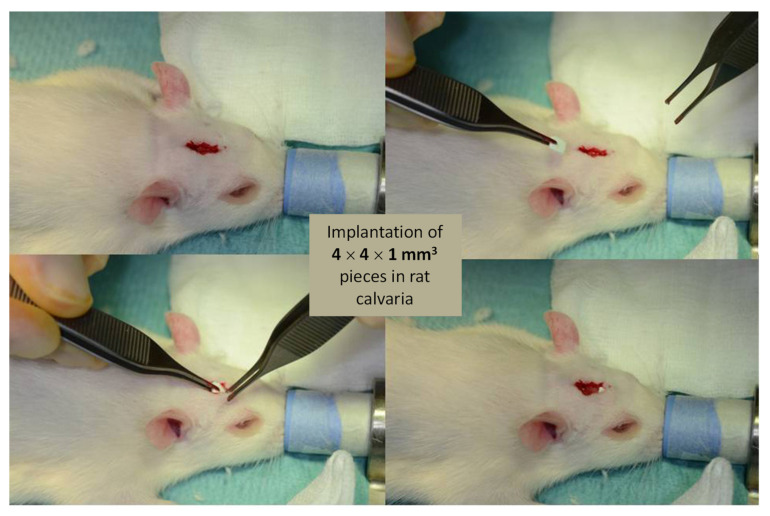
Implantation procedure on top of the rat’s calvarial bone.

Finally, a preliminary implantation study was performed in a calvarial defect in a rat to verify the good biocompatibility of such compounds in vivo. For this aim, the promising sample of hap-1d Ag 0.2% was selected as an illustrative example, taking into account its appealing antibacterial properties to both Gram-negative and Gram-positive bacteria. For comparative purposes, the non-doped hap-1d reference samples were also evaluated. The samples were pressed into pellets as reported above and finally cut into parallel piped 4 × 4 × 1 mm^3^ pieces to be positioned in the calvarial bone defect (Figure 11). Four rats were used in each test group.

**Figure 12:**
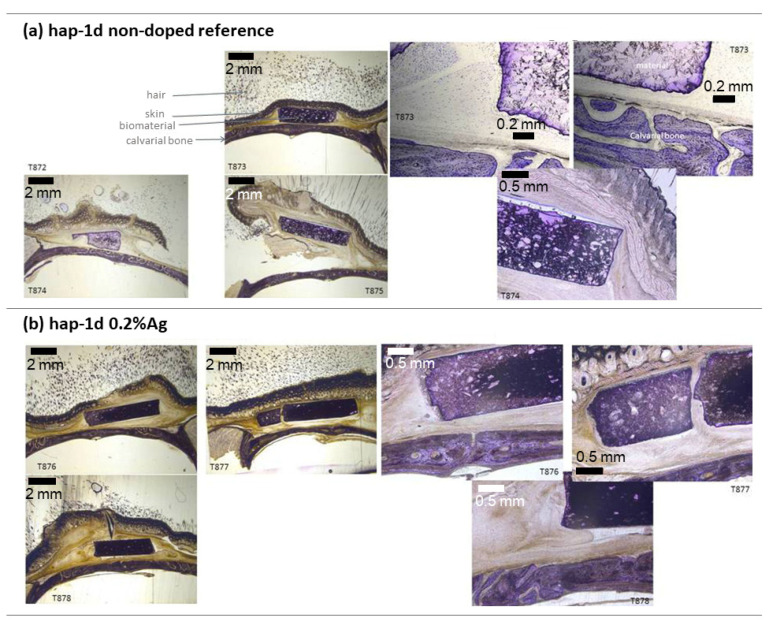
Histological images after implantation of hap-1d, non-doped (**a**), and doped (**b**) with 0.2% Ag. Images on the left provide general views and image on the right side provides higher magnification.

The placement of the materials was performed satisfactorily without any issues. All the implanted materials were well tolerated for the total implantation duration of 28 days since no adverse effects on the health of the animals were detected whatsoever following the operation. Histological sections were carried out on the explants to further study the local interaction with the biomaterials. The overview and higher magnification histological images are provided in Figure 12. No foreign body reaction could be detected in any of the histological section; only a layer of fibrous tissue was noted, for both types of samples, at the ceramic/calvarial bone interface (around 200 and 550 µm, respectively, for the non-doped and silver-doped samples).

This preliminary in vivo study indicates that the handling of the Ag-doped biomaterial was as good as the non-doped one. No sign of inflammation was detected in any of the ground sections. No adverse effects could be detected when the material was placed in close proximity to bone. Therefore, the materials appear biocompatible and reinforce the in vitro data reported above. In essence, these materials thus have the high level of biocompatibility needed for bone substitute materials. In addition, no difference in osteoconduction could be observed in this study between the non-doped and the Ag-doped sample, which behaved similarly.

## 4. Discussion

Biomimetic apatites are particularly suitable candidates for producing bone repair implantable materials due to their high similarity to bone mineral and their associated reactivity. However, the drying of apatite nanocrystals (e.g., in biomaterial production lines) leads to a supplementary surface amorphization associated with a partial disorganization of top-surface ions as we evidenced here. Upon (first) re-immersion in an aqueous medium of dried non-carbonated nanocrystalline apatites as produced in this work, this “additional surface amorphization” allows for releasing loosely-bound top-surface ions, which we identified as H_2_PO_4_^−^ and Ca^2+^ (in 2:1 proportions). In particular, the release of H_2_PO_4_^−^ ions into the solution can generate an acidification of the surrounding medium. This effect may prove transiently deleterious to the generalized production of biomaterials based on nanocrystalline apatites, depending on their conditions of preparation and actual characteristics, due to detrimental consequences of acidity on cells, and this effect could alter in vitro cell test results (mammalian or bacterial).

In this contribution, we clarified, for the first time, the reasons of this acidification phenomenon and, in cases where it needs to be circumvented, we then proposed a modification of the washing step involving an alkaline equilibration washing step prior to drying, which we validated using pH monitoring over 7 h of immersion in aqueous medium. By unveiling (and solving) this acidification issue and elucidating its origin, this work is intended to prove helpful for the future valorization of (non-carbonated) biomimetic apatites as genuine material-producing compounds for future advanced medicine. It can also help biologists to understand the surprising/perplexing results potentially noticed in some conditions in vitro when dealing with nanocrystalline apatites.

With this greater understanding of nanocrystalline apatite surface properties, the further development of biomimetic nanocrystalline apatites (non-carbonated) is expected to be facilitated in view of transfer to the industry and then the clinic. We showed in vitro that equilibrated apatite samples were not cytotoxic to human osteoblast cells and could be conferred as additional properties such as being antibacterial using relevant ion doping via Cu^2+^ or Ag^+^ ions. The antibacterial efficacy was also tested toward several bacterial strains of relevance to bone or dental infections. The Cu-doped biomimetic apatites proved to be antibacterial toward Gram-positive species and we report, for the first time, the reduction in bacterial colonies of *A. denticolens*, an anaerobic strain of relevance to dental infections. The Ag-doped samples were found to be active on both Gram-positive and Gram-negative bacteria relevant to bone infections. Both the Ag^+^ and Cu^2+^ doping ions used in this work to provide antibacterial properties to “equilibrated” nanocrystalline apatites may act in extracellular and intracellular ways. Indeed, the antibacterial properties of Ag^+^ were shown in the literature to be assignable to multiple mechanisms either necessitating or not an internalization inside the cells: Ag^+^ ions were reported to cause anomalies on the bacterial membrane [17,18,19], unveiling an external effect, but also in an intracellular way by interacting with the bacteria DNA [19,20] and enzymatic activity [21]. Similarly, the Cu^2+^ ions exhibit diverse antibacterial effects, both at the plasma membrane level by linking to membrane proteins [22,23] and internally by denaturing the bacteria’s DNA [22,24]. This is relevant to the present work as we may anticipate both “contact” and “distant” antibacterial action of the doping ions used here, which may explain our in vitro findings. In the case of a distant mode of action, the interaction with blood proteins and other chemical entities will also likely influence the activity. The Ag- or Cu-doped (nano)systems have already been discussed in the literature for diverse applications from bone repair to wound healing [16,20,21,24,25,26,27,28] and this duality of distant/contact effects appears to be very relevant in widening the treated area of the biological tissues in question.

A preliminary implantation study was finally performed in the rat calvaria to verify the good biocompatibility in vivo. Despite some degree of fibrous tissue formation, the biocompatibility of the two tested samples (non-doped and 0.2 mol.% Ag-doped biomimetic apatites) was confirmed without any detrimental reaction.

All the above findings show that such biomimetic apatite compounds, surface-equilibrated by following the protocol described here, are particularly relevant for the preparation of bioactive/bio-functional bone biomaterials.

## Data Availability

Data are available by contacting the corresponding author.

## Supplementary Materials

The following supporting information can be downloaded at: https://www.mdpi.com/article/10.3390/bioengineering10020220/s1, Figure S1: pH evolution over time at RT of deionized water, with re-immersed hap-1d or hap-20 min apatite compounds; Figure S2: pH evolution over time at RT of deionized water, with re-immersed stoichiometric HA (a) Ca_10_(PO_4_)_6_(OH)_2_ or apatitic TCP (b) (Ca_9_(PO_4_)_5_(HPO4)(OH) (with only apatitic HPO_4_^2−^ ions). As expected in these cases, no pH drop was observed in contrast to nanocrystalline apatites; Figure S3: Cytotoxicity evaluation (CAL-72 human osteoblast cells) of non-pre-equilibrated hap-1d reference sample (non-doped) in our working conditions relative to the controls. Neutral Red test. t = 24 h; Figure S4: Main physicochemical features (using XRD, FTIR, and atomic absorption spectroscopy) of Cu^2+^- and Ag^+^-doped apatite samples; Figure S5: Cytotoxicity evaluation (CAL-72 human osteoblast cells) of pre-equilibrated hap-1d samples doped with either Cu^2+^ (a) or Ag^+^ (b) ions in a wide range of doping rates (substitution of calcium in the apatite precipitation medium with the indicated percentage), in our working conditions, relative to the controls. Neutral Red tests; Table S1: ICP-OES measurements of the Ca- and P-released contents in solution upon the re-immersion of hap-20 min, hap-3 h, and hap-1d samples (water, initial pH 7.4). The Ca and P calibration lines led, respectively, to correlation coefficients R^2^ of 0.9998 and 0.9999. The mean relative error on each measurement is estimated as 2%.
