# Supplementary material for: Nanocrystalline Apatites: Post-Immersion Acidification and How to Avoid It—Application to Antibacterial Bone Substitutes
Source: Bioengineering (Basel). 2023 Feb 7;10(2):220. doi: 10.3390/bioengineering10020220 (PMC9952497; doi:10.3390/bioengineering10020220)
Supplement: Supplementary file 1 [file bioengineering-10-00220-s001.zip › bioengineering-2194443-supplementary.pdf]

"Nanocrystalline apatites: post-immersion acidification and how to avoid it. Application to antibacterial bone substitutes"

Drouet *et al.*

Supplementary Information

Figure S1. pH evolution over time at RT of deionized water, with re-immersed hap-1d or hap-20min apatite compounds.

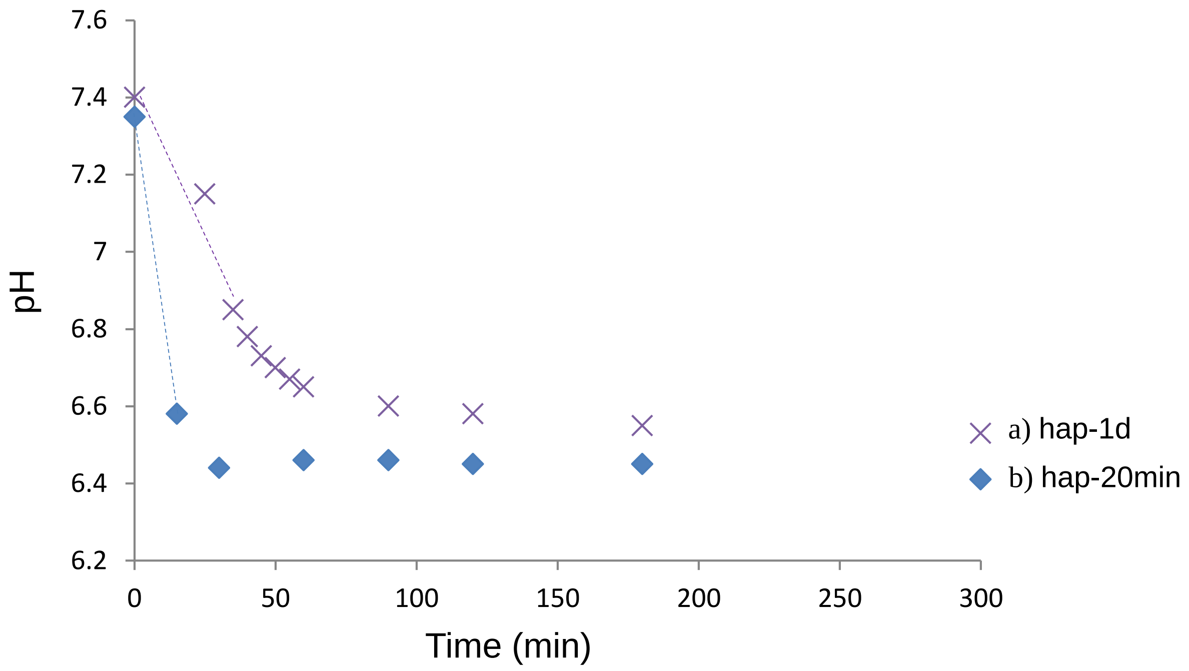

Figure S2. pH evolution over time at RT of deionized water, with re-immersed stoichiometric HA (a) $Ca_{10}(PO_4)_6(OH)_2$ or apatitic TCP (b) $(Ca_9(PO_4)_5(HPO_4)(OH)$ (with only apatitic HPO_4^{2-} ions). As expected in these cases, no pH drop was observed in contrast to nanocrystalline apatites.

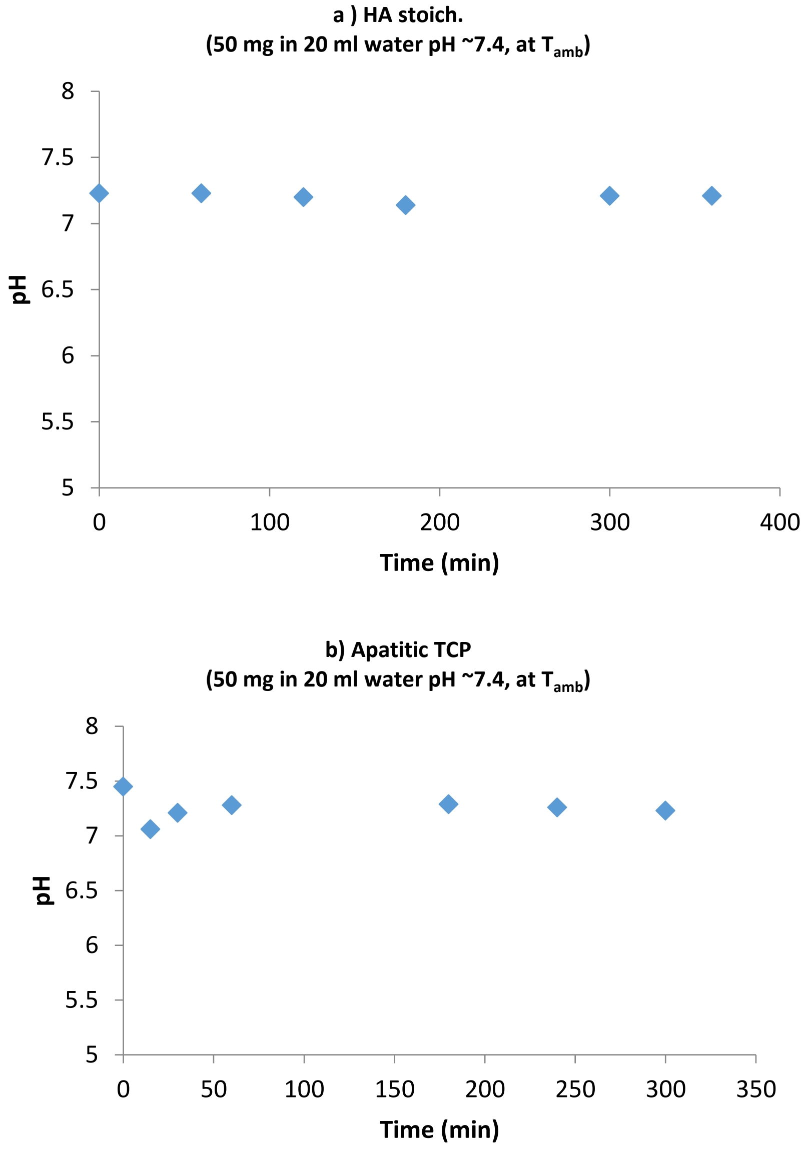

Figure S3. Cytotoxicity evaluation (CAL-72 human osteoblast cells) of non pre-equilibrated hap-1d reference sample (non doped) in our working conditions relative to the controls. Neutral Red test. t = 24 h

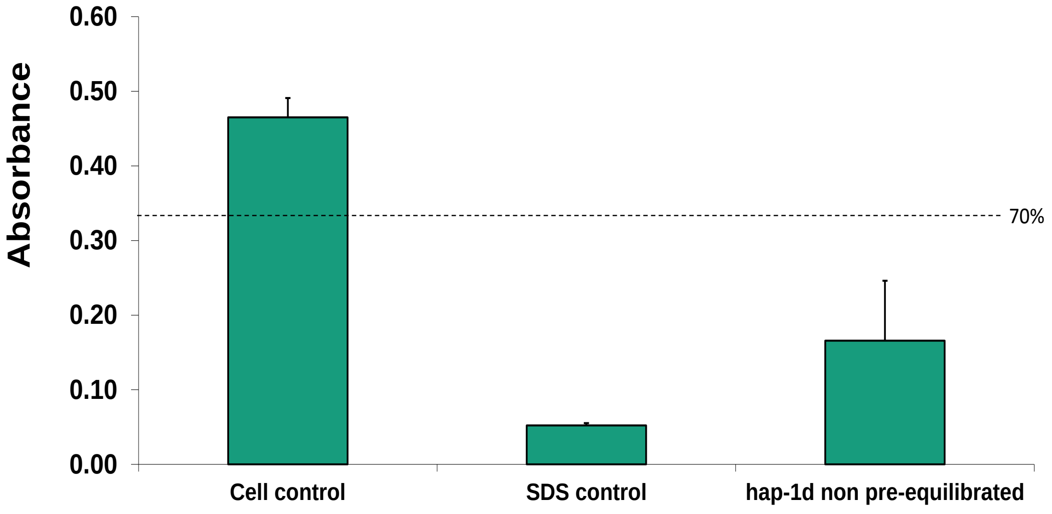

Figure S4. Main physicochemical features (through XRD, FTIR and atomic absorption spectroscopy) of Cu^{2+}- and Ag^{+}-doped apatite samples

XRD

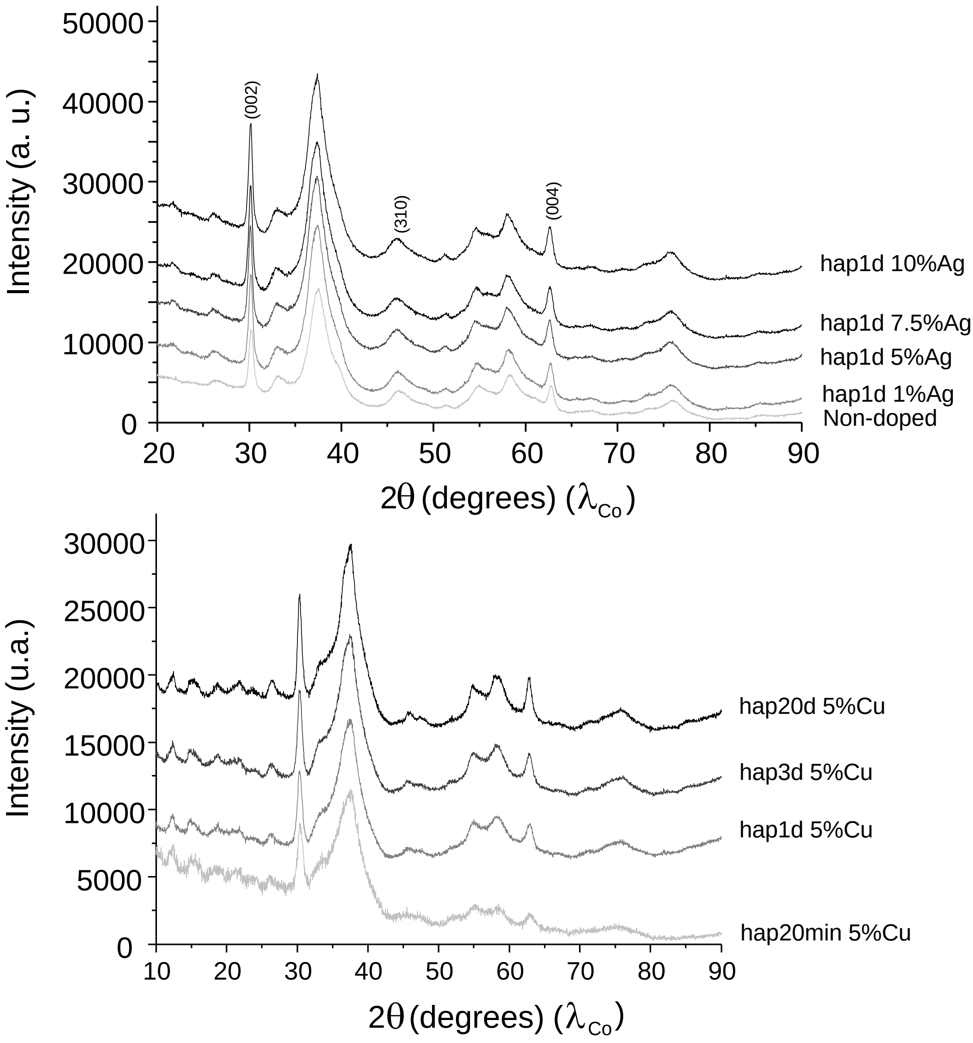

Atomic absorption spectroscopy

Sample	Initial Ag mol.% in solution	Final Ag mol.% in solid	(Ca+Ag)/P
hap1d	0	0	1.46 ± 0.02
hap1d 1%Ag	1.0	1.1	1.49 ± 0.02
hap1d 5%Ag	5.0	3.4	1.50 ± 0.02
hap1d 7.5%Ag	7.5	4.00	1.51 ± 0.02

Sample	Initial Cu mol.% in solution	Final Cu mol.% in solid	(Ca+Cu+Na)/P
hap3d	0	0	1.46 ± 0.02
hap3d 1%Cu	1.0	0.9	1.41 ± 0.02
hap3d 5%Cu	5.0	4.7	1.44 ± 0.02
hap3d 10%Cu	10.0	9.6	1.48 ± 0.02

Figure S4 (continued)

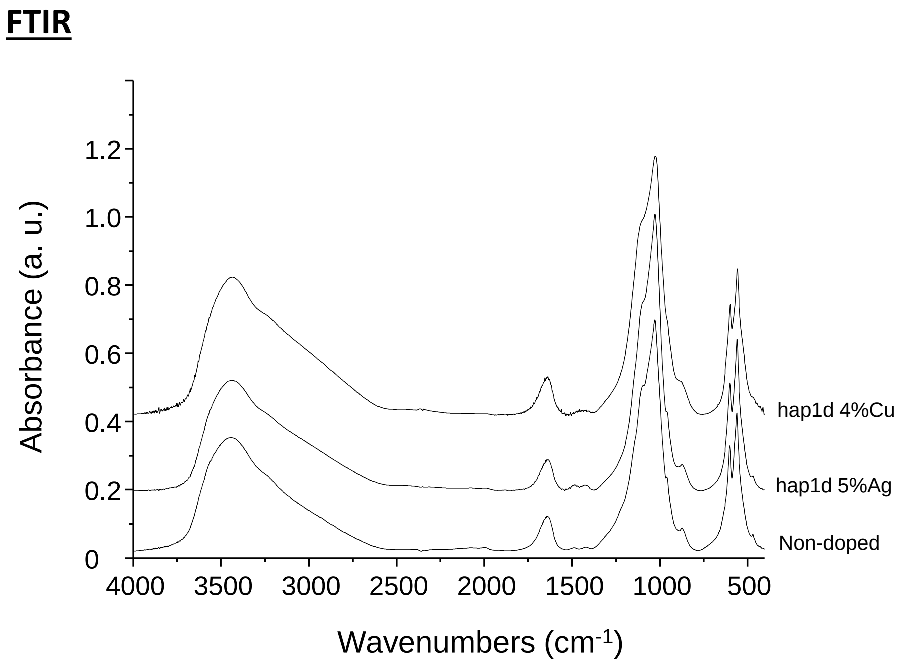

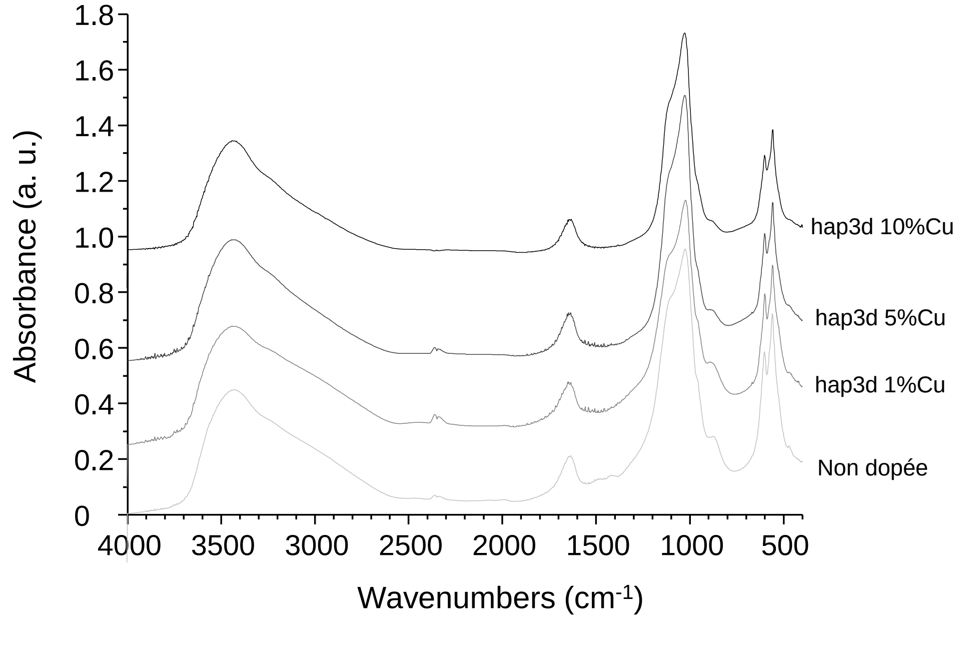

Figure S5: Cytotoxicity evaluation (CAL-72 human osteoblast cells) of pre-equilibrated hap-1d samples doped with either Cu^{2+} (a) or Ag^{+} (b) ions in a wide range of doping rates (substitution of calcium in the apatite precipitation medium with the indicated percentage), in our working conditions, relative to the controls. Neutral Red tests.

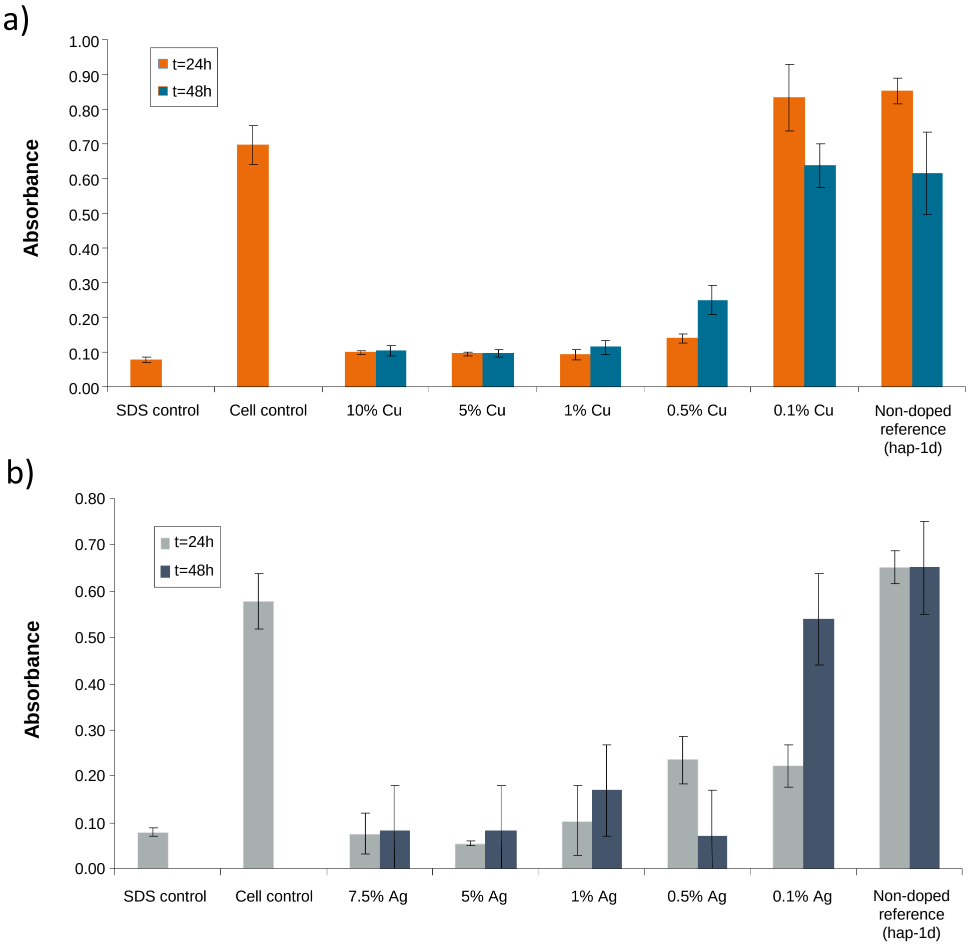

Table S1. ICP-OES measurements of the Ca and P released contents in solution upon re-immersion of hap20min, hap3h and hap1d samples (water, initial pH 7.4). The Ca and P calibration lines led respectively to correlation coefficients R^2 of coefficient de correlation 0.9998 and 0.9999. The mean relative error on each measurement is estimated to 2%.

Sample (50 mg in 20 ml)	Ca (ppm)	P (ppm)	molar Ca/P in solution
hap20min	23.87	39.73	0.47
hap3h	30.61	42.61	0.56
hap1d	18.50	32.38	0.44
